# Changes in health-related quality of life in people with morbid obesity attending a learning and mastery course. A longitudinal study with 12-months follow-up

**DOI:** 10.1186/1477-7525-10-95

**Published:** 2012-08-18

**Authors:** Randi Andenæs, May S Fagermoen, Hilde Eide, Anners Lerdal

**Affiliations:** 1 Dept of Nursing, Faculty of Health Science, Oslo and Akershus University College of Applied Sciences, Box 4, St. Olavs plass, 0130, Oslo, Norway; 2 Department of gastroenterology, Oslo University Hospital, Oslo and Institute of Health and Society, University of Oslo, Oslo, Norway; 3 Department of Health Sciences, Buskerud University College, Buskerud, Drammen; 4 Lovisenberg Diakonale Hospital, Oslo, Norway &Department of gastroenterology, Oslo University Hospital, Oslo, Norway

**Keywords:** Obesity, Health-related quality of life, Social support, Patient education, Bariatric surgery

## Abstract

**Background:**

Severe obesity is a complex condition that is associated with a wide range of serious health complications and reduced health-related quality of life (HRQoL). In addition to physiological factors, activity and participation, environmental factors, and personal factors are related to an individual’s overall quality of life HRQoL. In Norway, a course based on cognitive behavioral principles is offered to people seeking medical treatment for weight management. The aim is to assist participants to achieve a healthier lifestyle and thereby improve their HRQoL. We therefore investigated changes in HRQoL in participants after they attended this learning and mastery course, and explored how well sociodemographic variables, paid work, social support, personal factors, and surgery predicted HRQoL at 12-month follow-up.

**Methods:**

A single-group longitudinal study was conducted. Data were collected by self-reported questionnaires. This article reports on those who had completed the questionnaire at the 12-month (*n* = 69) follow-up. HRQoL was assessed with the EQ-5D. Other standardized instruments measured employment, social support, self-efficacy, and surgery.

**Results:**

At the 12-month follow-up, participants scored higher on all dimensions of the EQ-5D and on the EQ-VAS. Generalized linear model showed that having paid work, and social support were statistically significant predictors of HRQoL at the 12-month follow-up. Sex, self-efficacy, and surgery were not statistically significant associated with HRQoL.

**Conclusions:**

Participation in paid work, and receiving social support from persons with whom they had a close relationship were strongly related to HRQoL in obese people 12 months after participating in a learning and mastery course.

**Trial registration:**

The study is registered in Clinical Trials: NCT01336725.

## Background

All over the world, the prevalence of overweight and obese people is developing rapidly [1]. The World Health Organization’s Global Database on Body Mass Index indicates that the prevalence of obesity [body mass index (BMI)> 30 kg/m^2^ in the US adult population has reached 35.7% [2]. In Norway, the prevalence of 23.0% is alarmingly high [3]. Severe obesity is a complex condition affected by genetic, metabolic, social, behavioral, and cultural factors, and is associated with a wide range of serious health complications [4], and reduced health-related quality of life (HRQoL) [5].

The traditional goals of obesity treatment are to reduce body weight, maintain a lower body weight over the long term, and prevent further weight gain [6]. Because effective, sustained weight loss is not easily achieved, medical treatment, including surgery, is offered to morbidly obese people. Bariatric surgery is regarded as an effective weight-loss option for people whose obesity poses a major problem [7]. However, those seeking bariatric surgery report poorer quality of life than the obese in population studies [8]. It is therefore important to identify factors that may predict an improved quality of life for the morbidly obese who seek treatment.

In patients accepted for bariatric surgery, there is empirical evidence that a greater BMI [9], musculoskeletal pain and depression [10], and number of co-morbidities are risk factors of having lower overall HRQoL [9]. Even though women are more than five times more likely to seek bariatric surgery than men [11], the literature reveals conflicting results on HRQoL in obese people with respect to sex differences. In a Swedish study [12], women reported greater psychosocial problems in everyday life on an obesity-specific measure of HRQoL, but not in a generic one. Other studies examining sex differences in bariatric surgery patients found no differences [13,14]. In a recent study using survey data from a US national sample [15], both mental and physical HRQoL were worse in obese women, but only physical HRQoL was worse in obese men than in men of normal weight.

A literature review has emphasized the importance of increased activity as part of lifestyle behavior for patients undergoing bariatric surgery [16]. Participating in paid work represents an important type of activity, and two research group report on this topic. Andersen et al. (2010) found employment to be positively related to HRQoL in morbidly obese treated with duodenal switch surgery [17]. A study of 143 treatment-seeking morbidly obese reported that the physical and mental scores of the Short-Form-36 Health Survey (SF-36) were significantly higher in the employed group than the unemployed group [18]. HRQoL was assessed with the SF-36 in both studies.

The environmental factors of perceived social support, refers to the belief that help will be available from close persons if needed. In obese youth, perceptions of social support are significant predictors of overall HRQoL [19]. Although social support has rarely been studied in the adult obese population, Wiczinski et al. (2009) demonstrated that social support was significantly associated with better physical and mental HRQoL [20].

Personal factors comprise internal aspects such as self-efficacy, which may influence functioning. There is empirical evidence that self-efficacy is a possible mediator in improving lifestyle change, but not quality of life in obese people [21].

The effect of bariatric surgery in comparison with other approaches has been addressed. Adams et al. (2010) [22] studied three groups of severely obese people over 2 years (*n* = 1,156): gastric bypass patients, people seeking gastric bypass, and population-based obese people who were not seeking surgery. While HRQoL improved significantly in the surgery group, and in the control group with people not seeking surgery; those seeking bypass surgery had significant reductions in the mental component score of the SF-36.

Bariatric surgery also causes nutritional deficiencies and negative side effects, and recovery can be challenging, requiring a lifelong commitment to behavioral change, such as diet and activity [23]. In Norway, most hospitals have a Patient Education Resource Center that offers courses for patients with chronic illnesses or long-term ailments. The aims are to increase the participants’ knowledge about the consequences of their illness, and required lifestyle changes. At some centers, a 40-hour course is offered for morbidly obese patients awaiting treatment. The course is grounded in social cognitive theory [24] and emphasizes participants’ own work in uncovering unrecognized resources, strengthening self-concept, and coping skills, and improving the conscious consideration of lifestyle choices. The use of individual action plans and participation in self-help groups, are the core methods for improving HRQoL. Self-efficacy may be affected as a result of course participation. Providing more information on factors associated with HRQoL may facilitate further development of courses offered to the morbidly obese waiting for medical or surgical treatment.

The International Classification of Functioning, Disability and Health (ICF) framework [25] was used in the present study. According to this biopsychosocial framework, the meaning individuals attribute to their current body structure and functioning, activities they can or cannot engage in, and the degree to which they participate in society or are restricted from doing so reflects their HRQoL [26]. There is still a paucity of information about HRQoL and biopsychosocial factors in those who pursue obesity treatment. The inclusion of such participants in a comprehensive lifestyle course provided the opportunity to study changes in HRQoL over some time, and to explore predictors of overall HRQoL.

Therefore, this study aimed to: (1) examine the changes in HRQoL from baseline to12-months after course completion, and (2) explore associations between overall HRQoL and selected variables representing the ICF components; sex, paid work, social support, self-efficacy, and surgery, at 12-months follow-up.

## Methods

A single-group longitudinal study was conducted. Data were collected by means of 12 validated questionnaires from participants at learning and mastery courses. Three of the questionnaires were used in this study.

### Procedure

This was a study where patients with morbid obesity on the waiting list for bariatric surgery were given the option of continuing on the waiting list by enrolling in a mandatory educational lifestyle course. Participants were recruited at three different geographical sites on the first day of 10 courses held in the spring of 2009 in the eastern part of Norway. Data were collected at baseline and at 12-months after course completion. At baseline, the participants answered the questionnaire on the first or second day of the education course in a secluded room on-site and returned it in a sealed envelope. The project representative collected the envelopes. Data collection at the follow-up point was done by mailed questionnaires, using stamped and addressed return envelopes. One reminder was sent out to non responders. All 185 persons attending the course were given verbal and written information about the study and invited to participate;142 consented (77%). Course attendance was the only defined criterion. The mean age among those who consented (42.5 years, *SD* = 10.4) was not significantly different from the age of those who did not (*n* = 43, *M* = 44.2, *SD* = 9.1, *t* = −.98, *p* = .33). The proportion of women among the participants (70.4%) did not differ from the proportion in rest of the population (60.5%, *χ*^2^ 1.51, *p* = .22). At the 12-month assessment, 71 questionnaires were returned, and of these had 69 (48.6%) valid responses at baseline and 12-months follow-up. Missing scores in the indexes were tolerated up to 20% and were replaced by the individual’s mean response on the index.

### Measurements

#### HRQoL

To evaluate the participants’ HRQoL, we used *the EuroQol questionnaire (EQ-5D)*[27]. The EuroQol consists of two parts: a description of the person’s health status (EQ-5D) and a visual analogue scale (EQ-VAS), representing an overall measure of HRQoL [28]. The EQ-5D records the level of self-reported problems according to five dimensions; mobility, self-care, usual activities, pain/discomfort, and anxiety/depression. Each dimension is assessed using a single question with three response levels: 1 (no problem), 2 (some problems), and 3 (severe problems). The EQ-VAS is a single-index value, in which participants are asked to value their own health state ‘today’ on a 20 cm vertical visual analogue scale ranging from 0 to 100 (from worst to best imaginable health status respectively).

#### Sociodemographic characteristics

Data on age (years), sex, marital status (married/cohabiting versus not), and education level (≤12 years versus 13 years and more) were obtained.

#### Activities and participation

To measure employment status, data was coded as paid work or unpaid work.

#### Environmental characteristics

Social support was measured with the single item: ‘I think I have enough support from people with whom I have a close relationship’ [29]. A five-point Likert-type scale, ranging from ‘totally agree’ to ‘totally disagree’ was used for scoring the participants’ answers. High scores indicated a ‘very satisfied’ assessment of the support received.

#### Personal factors

Self-efficacy was measured with the *General Perceived Self-Efficacy Scale (GSE)*[30]. General self-efficacy refers to the belief in one’s competence to cope with stressful or challenging demands. The scale consists of 10 items rated on a four-point scale with the anchors from ‘completely disagree’ to ‘completely agree (range: 10–40). A GSE score is calculated by summing each individual’s scores for the items, with higher scores indicating high general self-efficacy. Internal consistency (Cronbach’s *α*) in this study was .93.

#### Health condition

At the 12-month follow-up, the participants were asked whether they had undergone surgery or conservative treatment; the response alternatives were ‘yes’ and ‘no’.

### Ethics

The Regional Medical Research Ethics Committee of Norway (REK S-08662c 2008/17575) and the Ombudsman of Oslo University Hospital approved the study. Informed written consent was received from all participants.

### Statistical analysis

Data were analyzed using PASW for Windows (version 18.0; SPSS Inc., Chicago, IL). Independent and paired-samples *t-*tests were used to analyze continuous variables. Ordinal and categorical data were analyzed using chi-square (*χ*^*2*^) and Fisher’s exact test. Cronbach’s *α* was performed on baseline data to assess the internal consistency of the scales.

To determine the predictive values of the independent variables for HRQoL, we used GLM (generalized linear models) for repeated measures with EQ-VAS scores at baseline and at 12-month follow-up as the outcome. Predictors were entered into the equation according to the theoretical model of the study, which represented the four components of the ICF [25], and the following predictor variables were included in the analyses: *Body functions and structures,* sex; *Activities and participation,* work status; *Environmental factors*, social support; *Personal factors*, self-efficacy. The general rule recommends that about 15 participants per predictor variable are needed for a reliable model. Because of the high attrition rate and therefore low sample size (n = 69) in the study, the greatest number of predictors in the GLM was restricted to four. This calculation of predictors indicated that a minimum sample size of 60 participants was necessary. The level of significance was set at *p*< .05. All tests were two-tailed. Cohen’s *d* was used to calculate effect size [31].

## Results

### Attrition analyses

The mean age of the participants with complete data at 12-month follow-up (43.4 years, *SD* = 10.2) did not differ from that of participants from the baseline sample who consented but were excluded because of missing scores (*n* = 73, *M* = 41.6 years, *SD* = 10.6, *t* = −1.03, *p* = .31). The proportion of women in the study sample (*n* = 51, 73.9%) did not differ from those with missing scores (*n* = 49, 67.1%, *χ*^*2*^ = .78, *p* = .46). Significant differences were revealed with employment status, where 63.8% of the participants in the study group were in paid work, compared with 40.3% (*χ*^*2*^ = 7.79, *p* = .01) in those who were excluded.

When we compared these groups with regard to their respective scores at baseline, we found no statistical differences in self-efficacy or EQ-VAS scores (data not shown).

### Sample description

Baseline characteristics of sociodemographic variables and personal factors of the sample are presented in Table 1. The average age of the participants was 43.7 years and 73.9% were women. There were no sex differences on any baseline characteristics of the participants. At 12-month follow-up, 72.5% (n = 50) had undergone bariatric surgery, and 7.5% (n = 5) conservative treatment. Fourteen participants did not report on the health condition question.

**Table 1:** Baseline characteristics of the study participants

	**All**	**Men**	**Women**		
	***N*= 69**	***N*= 18**	***N*= 51**		
	***M*(*SD*)**	***M*(*SD*)**	***M*(*SD*)**	***t***	***p-value***
Age (years)	43.4 (10.2)	44.4 (9.0)	43.0 (10.7)	.50	.62
Social support					
(scale 1 – 5)	4.0 (.9)	4.0 (.8)	4.0 (1.0)	.00	1.00
Self-efficacy					
(GSE scale 1 – 40)	26.5 (6.7)	28.0 (6.5)	26.0 (6.7)	1.06	.29
	*N* (%)	*N* (%)	*N* (%)	*χ*^*2*^ (*df*)	
*Level of formal education*				.53 (1)	.57
7–12 years	45 (65.2)	13 (72.2)	32 (62.7)		
13–years or more	24 (34.8)	5 (27.8)	19 (37.3)		
Married/cohabitating	47 (69.1)	15 (83.3)	32 (64.0)	2.32 (1)	.15
Paid work	45 (65.2)	15 (83.3)	30 (58.8)	3.52 (1)	.09
Bariatric surgery	50 (72.5)	11 (61.1)	39 (76.6)	1.58 (1)	.23

Note. Independent-samples *t*-test and chi-square tests are performed in order to assess possible differences between men and women.

### Health related quality of life

#### HRQoL at baseline compared with 12-month follow-up

**Figure 1:**
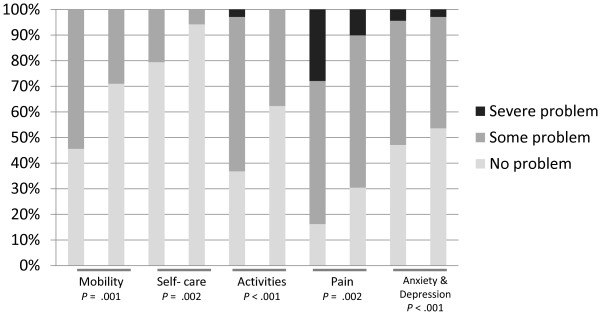
**Scoring (in proportions) of the EQ-5 sub-domains at baseline and 12-month follow-up (n = 69)**. Differences between the two time points are analyzed using chi-square and Fisher’s exact tests.

The participants showed improved overall HRQoL between baseline (*M* = 47.5, *SD* = 21.0) and 12-month follow-up (*M* = 67.7, *SD* = 20.6, *t* = −6.5, *p* < .001, *d* = .97). The improvement was significant for both women (*t* = −4.8, *p* = < .001) and men (*t* = −4.8, *p* = < .001). The chi-square tests showed that the participants reported statistically significant changes in mobility, self-care, activities, pain/discomfort, and anxiety/depression during the 12 month follow-up (Figure 1).

When the overall HRQoL scores of participants who had undergone bariatric surgery at 12-month follow-up (*n* = 50, *M* = 69.3, *SD* = 18.4) were compared with those who had not (*n* = 19, *M* = 61.2, *SD* = 25.3), the statistical test did not reach significance (*t* = −1.47, *p* = .08) but the effect size was clinically significant (*d* = .37). Those who had undergone surgery had a (not significant) tendency to report lower overall HRQoL scores at baseline (*M* = 46.1, *SD* = 22.3) than those who did not have surgery (*M* = 50.2, *SD* = 16.3, *t* = .72, *p* = .47, *d* = .21). Overall HRQoL among those who underwent bariatric surgery was higher at 12-month follow-up (*M* = 69.3, *SD* = 18.4) than baseline (*M* = 46.1, *SD* = 22.3, *t* = −6.3, *p* < .001). Participants who did not receive bariatric surgery also showed a significant tendency to have higher scores at 12-month follow-up (*M* = 61.2, *SD* = 25.3) than at baseline (*M* = 50.2, *SD* = 16.3, *t* = −2.1, *p* = .05). No statistically significant differences between the two groups were found on any other study variable neither at baseline nor at the 12-month follow-up.

#### Predictive model for HRQoL

To adjust for baseline levels of EQ-VAS we used GLM. Self-efficacy was not statistically significant associated with EQ-VAS so we omitted it from the final model. We fitted GLM for repeated measures. Work and social support remained statistically significant (*p* = .005 and *p* = .026 respectively) when adjusted for sex and surgery.

## Discussion

This prospective study of a population of participants taking part in learning and mastery courses demonstrated significant improvement in HRQoL after 12 months. Improvements were seen in all dimensions of the EQ-5D. Further, participating in paid work, and receiving social support were significant predictors of overall HRQoL at 12-month follow-up, while self-efficacy was not predictive of HRQoL.

Being in paid work seems to be an important predictor of HRQoL, and the positive association between paid work and HRQoL in this population corresponds with the findings of previous research [17,18]. The association is most likely to be bidirectional: It is possible that not having a paid job is primarily a consequence of being severely obese and having poor HRQoL. Morbid obesity has been associated with decreased participation in paid work, covering increased absenteeism and disability [32]. The reasons for decreased employment in paid work are assumed to be either a result of stigmatization of obese people in our Western culture, which leads to discrimination in the labour market, or because health reasons limit the ability of obese people to work [33]. A possible explanation for the positive association with HRQoL is that a person’s work role is regarded as their major social identity in most contemporary post industrial societies. An important aspect of employment is that working usually imposes an organized structure on person’s life and provides a social world that is different from the family and household social network [34]. Bariatric surgery improves the chance of participating in the labour market.

Receiving social support from close persons was also a significant predictor of HRQoL in our study. Our findings support those of Wiczinski et al. [20], who reported social support to be significantly associated with better physical and mental HRQoL. According to the buffering model of support, social support is beneficial because it decreases the negative effects of stress on both mental and physical health [35]. At the start of the course, our study participants were all on the waiting list for bariatric surgery. It is possible that this period of time before treatment was offered was perceived as stressful, and people who they could count on for help and support represented a key to improved HRQoL. In classic writings on stigma, Goffman (1963) observed that stigmatized individuals, including overweight and obese persons, may choose purposely to interact with others who also carry the stigma, and may offer one another acceptance, emotional and moral support, and empathy [36]. Consequently, provision of support in behavioral courses for morbidly obese people can lead to the formation of new relationships, such as self-help groups. Enhanced perceptions of social support are associated with adherence to exercise behaviors and attendance in exercise settings for healthy populations [37]. Such findings raise the question whether self-help groups and strengthened social ties also might help morbidly obese people who encounter numerous lifestyle adjustments associated with treatment, and in turn influence HRQoL.

We could not identify self-efficacy as a possible predictor of HRQoL. In the self-efficacy theory, a belief in one’s ability to exercise control over life is essential [24]. Other studies have found that self-efficacy increased after intervention [21], and it is possible that self-efficacy was outweighed by the effect of surgery with our participants. Bariatric surgery is commonly regarded as the definitive solution in overweight and obese people, and it could be that the prospect of undergoing bariatric surgery led patients to place responsibility for a cure on the medical team rather than on themselves [38].

The finding that participants who did not undergo surgery also demonstrated improved overall HRQoL, could indicate a separate effect of the mastery course; however, as the study was a descriptive, rather than an intervention study, no causal assumptions can be made. Positive changes in HRQoL are seen in experimental designs even in patients with no interventions [22].

The factors identified as associated with HRQoL could be helpful for health care providers that educate and counsel the morbidly obese before bariatric surgery. A comprehensive multidisciplinary program that incorporates biopsychosocial aspects may be of critical benefit in enhancing compliance, outcome, and quality of life in those seeking to lose weight [39]. Further investigation of HRQoL and factors that maintain and increase HRQoL for persons with morbid obesity is warranted.

### Strengths and weaknesses

The strengths of our study are the longitudinal design and the inclusion of social and personal variables that have rarely been studied. However, several limitations must be addressed. The absence of objective measures such as weight and BMI prevented us from controlling for this variable. However, previously published studies report no or only a weak relationship between BMI and mental components of HRQoL in obese people [40,41]. Further, a body weight procedure for the participants was considered to put emphasis on dieting above lifestyle choices, and thus interfere with the intended psychosocial learning processes.

A further limitation might be that social support was assessed by the exclusive reliance on participants’ self-rating on a single item (i.e., evaluation of support from close relationships). Subsequent studies could include more comprehensive measures of social support.

To motivate patients for follow-up was difficult, despite one remainder, and consequently, we lack complete data on 51% of the participants at 12-month follow-up. Low response rates are common in most longitudinal studies with self-reported questionnaires [28], and high rates of attrition are also reported in longitudinal obesity studies [42]. Attrition analyses found that our participants reported higher participation in paid work than those with missing scores, but no other differences were, however, found between the two groups. Thus, it seems reasonable to assume that findings in the present study are representative of the healthiest of those who seek medical treatment for severe obesity. It has been suggested that the effect of obesity on HRQoL might be a main reason to seek treatment [43]. Consequently, morbidly obese who are on the waiting list for treatment may be a self-selected sample with reduced HRQoL, and participants in the present study might not be representative of all obese persons. Despite these possible shortcomings, our results contribute to the limited body of research and knowledge of factors predicting HRQoL in morbidly obese people who seek treatment.

## Conclusions

HRQoL improved significantly at the 12-month follow-up assessment after attending a learning and mastery course. During the follow-up period, nearly three out of four participants underwent bariatric surgery. HRQoL also improved for participants who did not undergo bariatric surgery. We acknowledge that the causal mechanisms for the observed improvements in HRQoL may be multifaceted; the findings indicate that dynamics between environmental factors, participation in paid work, surgical and lifestyle interventions such as the learning and mastery course had a positive effect on HRQoL. The lack of a comparison group prohibits the exploration of causal relationships, so no definite conclusions can be drawn.

## Abbreviations

EQ-5D, EuroQol; GSE, General Perceived Self-Efficacy Scale; HRQoL, Health-related quality of life.

## Competing interests

The authors declare that they have no competing interests.

## Authors’ contributions

R A: wrote and revised the manuscript, participated in the design of the study and participated in the statistical analysis. M S. F: was the main contributor to the concept and design of the study, supervised the research group, collected the data, and critically revised and commented on the manuscript. H E: participated in the design of the study and commented on the manuscript. A L: contributed substantially to the concept and design of the study, performed the statistical analyses and drafted the manuscript. All authors read and approved the final manuscript.
